# Current Updates on Oxazolidinone and Its Significance

**DOI:** 10.1155/2012/159285

**Published:** 2012-02-26

**Authors:** Neha Pandit, Rajeev K. Singla, Birendra Shrivastava

**Affiliations:** ^1^Department of Pharmaceutical Chemistry, School of Pharmaceutical Sciences, Jaipur National University, Jagatpura-Jaipur, Rajasthan 302025, India; ^2^Sadbhavna College of Management & Technology, Jalaldiwal, Ludhiana-Barnala State Highway-13, Raikot (Ludhiana), Punjab, India

## Abstract

Oxazolidinone is a five-member heterocyclic ring exhibiting potential medicinal properties with preferential antibacterial activity. Scientists reported various synthetic procedures for this heterocyclic structure. Current review articles tried to cover each and every potential aspect of oxazolidinone like synthetic routes, pharmacological mechanism of action, medicinal properties, and current research activities.

## 1. Introduction

The oxazolidinones are a new class of antimicrobial agents which have a unique structure and good activity against gram-positive pathogenic bacteria. Oxazolidinones are a class of compounds containing 2-oxazolidine in the structure. Oxazolidinones represent a new class of synthetic antibacterial agents active against multiple-resistant gram-positive pathogens, including methicillin-resistant *Staphylococcus aureus* (MRSA), penicillin-resistant streptococci, and vancomycin-resistant enterococci [1].

### 1.1. Chemical Structure [2]

**Figure 1:**
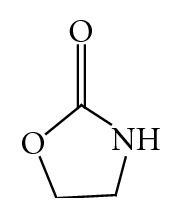
General Structure of oxazolidinone.

Oxazolidinones are a class of azoles, oxazolidines with the carbon between the nitrogen and oxygen oxidized to a ketone, hence oxazolidinone (Figure 1).

### 1.2. Nomenclature

The antibacterial oxazolidinone template has a common nomenclature specially for aryl-5-(substituted) methyl-2-oxazolidinone. Throughout the paper, a consistent numbering system has been employed for description of the various oxazolidinone examined [3].

## 2. Synthetic Schemes

Earlier reviews have comprehensively covered the many synthetic approaches available for construction of the oxazolidinone ring. The most recent literature is replete with accounts of oxazolidinone templates that have seen extensive use as chiral auxiliaries.

**Figure 2:**
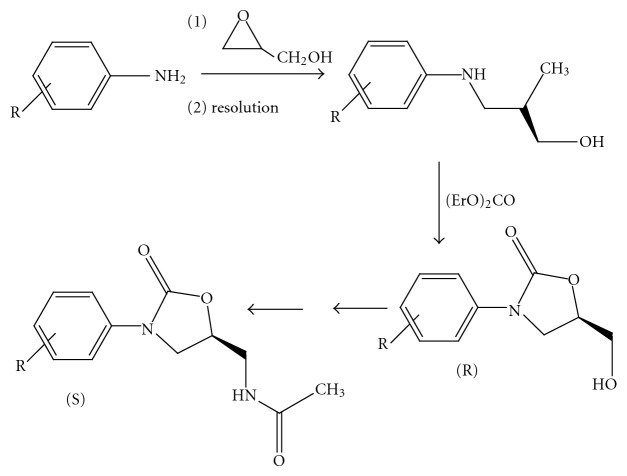
Chiral resolution method.

**Figure 3:**
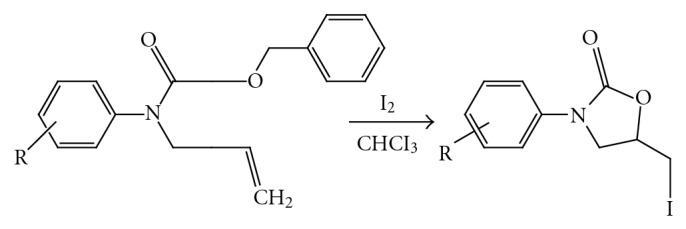
Iodocyclocarbamation.

**Figure 4:**
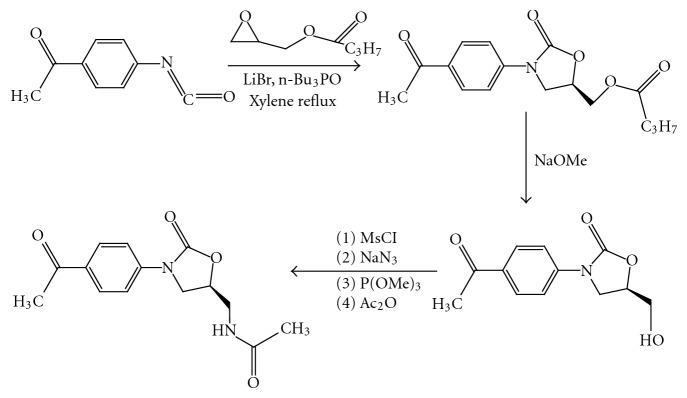
Du PONT asymmetric synthesis.

 (1) *Chiral Resolution Method.* Early work relied upon chiral resolution as a means to optically active oxazolidinone. The amino-diol resulting from reaction of an aniline with glycidol was resolved using (R)-mandelic acid. Diethylcarbonate effected cyclization to the 5-(R)-hydroxymethyl-3-phenyl-2-oxazolidinone. Numerous approaches had been employed for converting the 5-(R)-methylalcohol moiety to the 5-(S)-acetamidomethyl group. Here tosylation of the alcohol was followed by azide displacement, reduction, and acylation of the amine [4] (Figure 2). 

 (2) *Iodocyclocarbamation.* Many of the Upjohn SAR studies were carried out with racemic oxazolidinone, synthesized by iodocyclocarbamation. A key modification was the addition of pyridine, crucial for circumventing untoward alkylative side reaction [5] (Figure 3).

(3)* DuPONT Asymmetric Synthesis. The Herweh-Kauffmann/Speranza-Peppel Method.* This method involved high-temperature catalyst-mediated cyclization of an aryl isocyanate with an epoxide where LiBr catalyst was solubilized in refluxing xylene by tributylphosphine oxide. As demonstrated for DuP721 (R)-glycidol butyrate was cyclized with 4-acetylphenyl isocyanate, giving the oxazolidinone butyrate ester. Saponification provided the 5-(R)-hydroxymethyl oxazolidinone which was further methylated and then converted to the acetamide, as described next for tosylate [5] (Figure 4).

**Figure 5:**
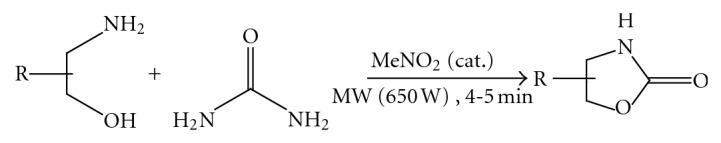
Oxazolidinones using urea and ethanolamine.

**Figure 6:**
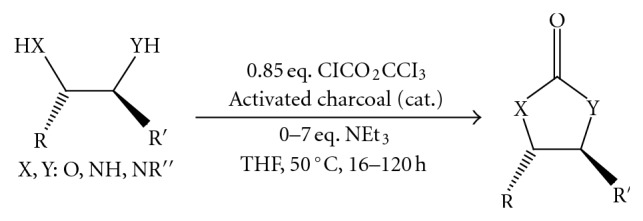
Efficient synthesis of oxazolidinone, imidazolidinone, and dioxolane.

**Figure 7:**
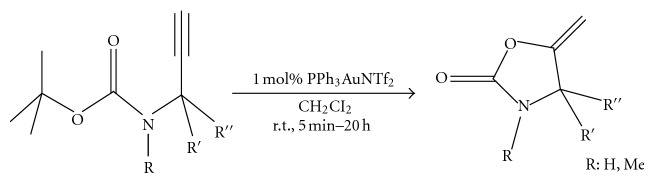
Gold catalyzed rearrangement in oxazolidinone synthesis.

**Figure 8:**
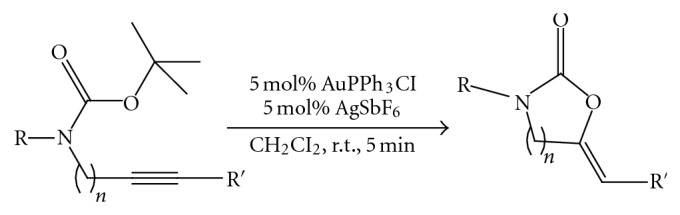
N-Boc protected oxazolidinone synthesis.

**Figure 9:**
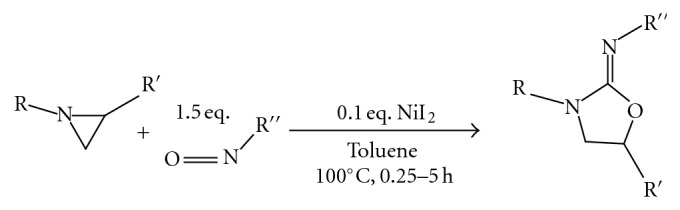
Nickel catalyzed cycloaddition in oxazolidinone synthesis.

**Figure 10:**
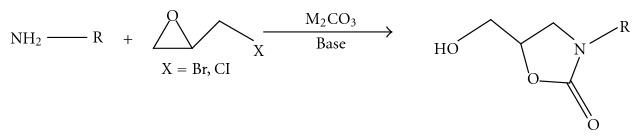
Use of halomethyloxirane, primary amine, and carbonate salt in oxazolidinone synthesis.

(4) Synthesis of oxazolidin-2-ones derivatives was carried out starting from urea and ethanolamine reagents using microwave irradiation in a chemical paste medium in which a catalytic amount of nitromethane absorbed the microwaves and generated hot spots [6] (Figure 5).

(5) An efficient, versatile, and practical gram-scale preparation of oxazolidinone, imidazolidinone, and dioxolanone was achieved [6] (Figure 6).

(6) A mild and efficient gold (I)-catalyzed rearrangement of propargylic *tert*-butylcarbamates allowed the synthesis of various 5-methylene-1, 3-oxazolidin-2-ones, which would be less conveniently obtained using other methods [7] (Figure 7).

 (7) Various *N*-Boc-protected alkynylamines were converted into the corresponding alkylidene 2-oxazolidinones or 2-oxazinones under very mild reaction conditions in the presence of a cationic Au (I) complex in high yield regardless of the substitution at nitrogen and alkyne terminus [8] (Figure 8).

(8) A nickel-catalyzed cycloaddition of aziridines with isocyanates proceeded smoothly to give iminooxazolidine derivatives in good yields. A longer reaction time allowed the isomerization of the iminooxazolidine to the corresponding imidazolidinone derivatives [9] (Figure 9).

(9)* Synthesis of Oxazolidinones by the Use of Halomethyloxirane, Primary Amine, and Carbonate Salt*. Primary amines reacted with carbonate salts (Na_2_CO_3_, K_2_CO_3_, Cs_2_CO_3_, and Ag_2_CO_3_) and halomethyloxiranes in the presence of a base such as DBU or TEA to give oxazolidinones in high yields. The use of K_2_CO_3_ among these carbonate gave the best yield in this synthesis. A reaction mechanism was proposed that the oxazolidinone was obtained from an oxazinanone intermediate via a bicyclo [2.2.1] intermediate. The present reaction can be widely applied to convenient synthesis of useful N-substituted oxazolidinones and chiral oxazolidinones [10] (Figure 10). 

**Figure 11:**
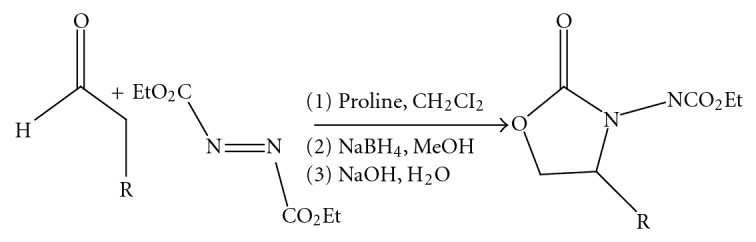
Direct catalytic asymmetric amination of aldehydes: synthesis of Evans, oxazolidinones and *α*-amino acids.

**Figure 12:**
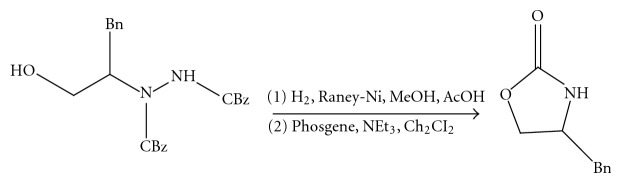
Conversion of chiral *α*-hydrazino alcohols and N-amino oxazolidinone to evans auxiliaries.

**Figure 13:**
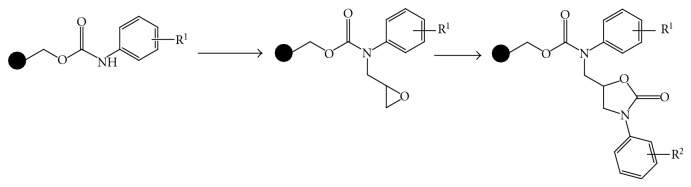
Solid-phase synthesis of oxazolidinones.

**Figure 14:**
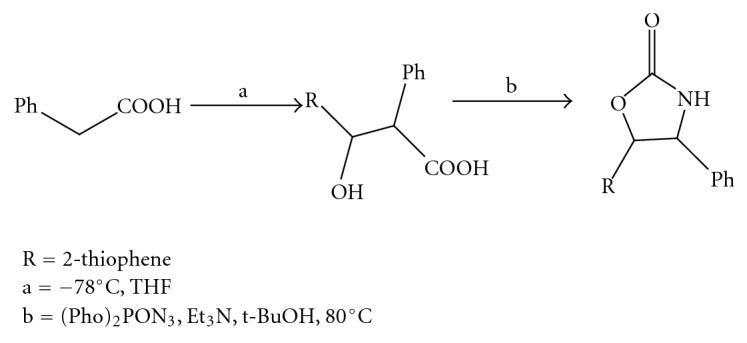
Diastereoselective synthesis of oxazolidinone part-I.

**Figure 15:**
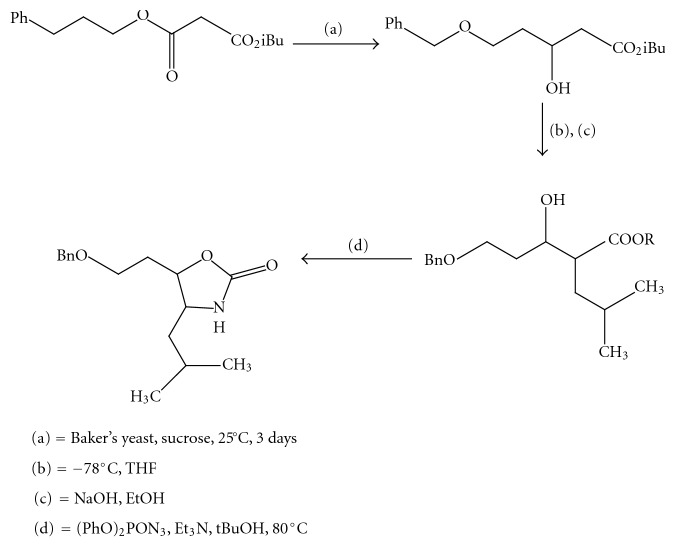
Diastereoselective synthesis of oxazolidinone part-II.

**Figure 16:**
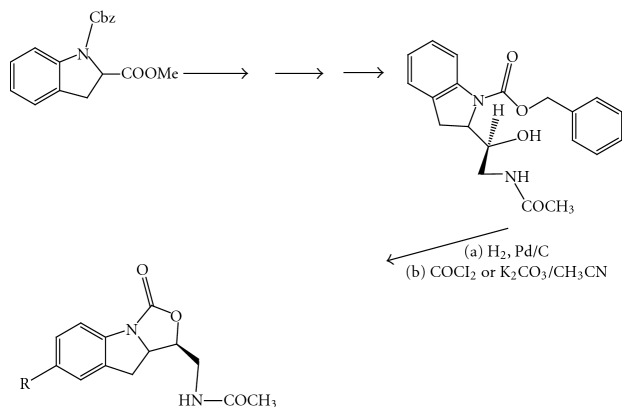
Diastereoselective synthesis of oxazolidinone part-II.

 (10) *Direct Catalytic Asymmetric Amination of Aldehydes: Synthesis of Evans, Oxazolidinones and *α*-Amino Acids *[11] (Figure 11). 

 (11) *Conversion of Chiral *α*-Hydrazino Alcohols and N-Amino Oxazolidinone to Evans Auxiliaries [11](Figure 12).*

(12) *Solid-Phase Synthesis of Oxazolidinones by Cycloaddition of Resin-Bound Epoxides with Isocyanates. *The first solid-phase synthesis of oxazolidinones by cycloaddition of resin-bound epoxides with isocyanates is described. Synthesis of the title compounds was achieved by alkylation of resin-bound carbamates with glycidyl tosylate, followed by cycloaddition of the resulting epoxides with isocyanates at elevated temperature in high yields and purity. Because *N*-aryloxazolidinones have been known to possess various biological activities, this method is useful from the viewpoint of drug discovery [12] (Figure 13).

(13) *A Convenient Diastereoselective Synthesis Of Oxazolidinone: Approach to Unusual Amino Acid Statine *[13] (Figures 14 and 15). 

(14) *Synthesis of *[5, 14, 14]*-Tricyclic Fused Oxazolidinone [15] (Figure 16).*

## 3. Pharmacological Background

**Figure 17:**
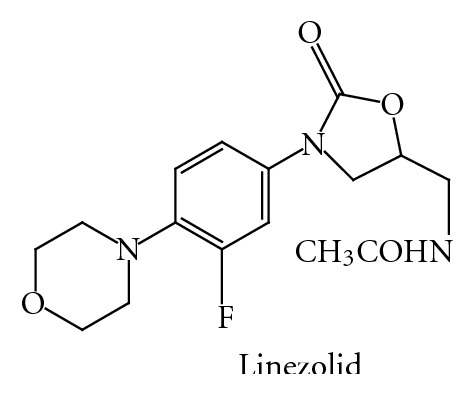


*Mechanism of Action*. Oxazolidinones inhibit protein synthesis by binding at the P site at the ribosomal 50S subunit. Resistance to other protein synthesis inhibitors does not affect oxazolidinone activity; however rare development of oxazolidinone resistance cases, associated with 23S r-RNA alterations during treatment, has been reported. Linezolid (Figure 17), the first oxazolidinone available, has already taken its place in the clinic for treatment of gram-positive infections.

**Figure 18:**
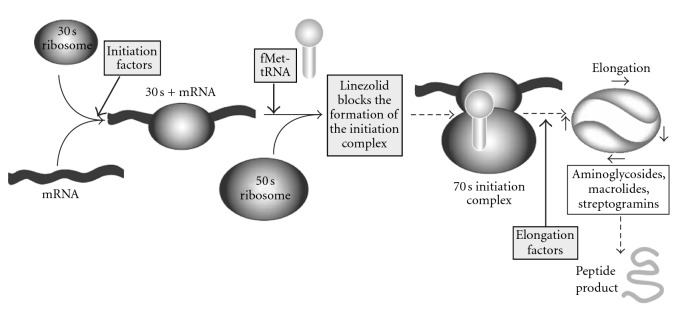


It selectively inhibits bacterial protein synthesis through binding to sites on the bacterial ribosome and prevents the formation of a functional 70S-initiation complex. Specifically, linezolid binds to a site on the bacterial 23S ribosomal RNA of the 50S subunit and prevents the formation of a functional 70S initiation complex (Figure 18), which is an essential component of the bacterial translation process [16].

## 4. Pharmacokinetic Parameter [17, 18]

- *Absorption*. It is rapidly and extensively absorbed after oral dosing. Maximum plasma concentrations are reached approximately 1 to 2 hours after dosing, and the absolute bioavailability is approximately 100%.
- *Toxicity*. Clinical signs of acute toxicity lead to decreased activity, ataxia, vomiting and tremors.
- *Protein Binding*. 31%.
- *Biotransformation.* It is primarily metabolized by oxidation of the morpholine ring, which results in two inactive ring-opened carboxylic acid metabolites: the aminoethoxyacetic acid metabolite, and the hydroxyethyl glycine metabolite.
- *Half-Life.* 4.5–5.5 hours.
- *Excretion*. Renal and fecal (Table 1).

### 4.1. Available Marketed Formulation [19]

#### 4.1.1. Bacterial Resistace and the Search for Novel Antibacterial Structural Templates [5]

- The alarming escalation seen worldwide in the incidence of bacterial resistance to previously effective antibiotics continues to provide the impetus for the medicinal chemist to search for entirely new classes of antibacterial agents, that can cure bacterial infection by novel mechanism. As numerous bacteria have increasingly evidenced the evolution of multiple-antibiotic resistance, health care provides have been seriously challenged to provide effective therapy for the often life-threatening infection caused by these pathogens. Numerous reviews have recently appeared emphasizing the extent and severity of the resistance problem as it exists today, and the dim prospects envisioned for the future.
- Some of the most problematic organism has been the multi-drug-resistant gram-positive bacteria. These include the highly virulent organism, methicillin-resistant *Staphylococcus aureus* MRSA, and penicillin-and cephalosporin-resistant *Streptococcus pneumoni. *
- The enterococci have become increasingly of concern; there have been isolated problems of infection with vancomycin-resistant enterococci VREae. A major concern is that VRE will transfer the vancomycin-resistance genes encoded on a plasmid, to much more virulent organism *S. aureus,* as Nobel et al. have demonstrated to be feasible in an experimental setting.

Still another problem microorganism for which resistance has generated considerable medical concern is multidrug-resistant *M. tuberculosis* MDRTB.

This loss of antibacterial activity among drugs once efficacious against these pathogens has led to a summoning from numerous experts for the discovery and development of new antibiotic classes, so that health care practitioners will not be left benefit of effective therapeutic modalities. The oxazolidinone antibacterial agents are capable to provide an answer for these problems in new age. 

## 5. Bacteriostatic versus Bactericidal Nature of Oxazolidinones [20]

**Figure 19:**
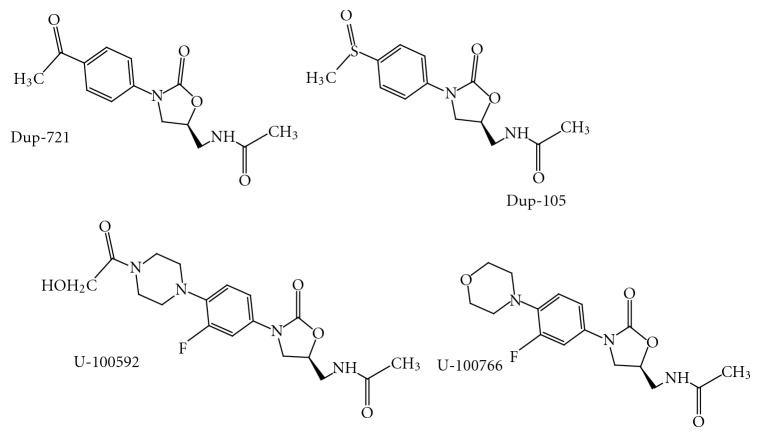


Time-kill studies indicate that both Dup-721 (Figure 19) and Dup-105 (Figure 19) were bacteriostatic against all organism tested, expect the diphtheroids but U-100592 (Figure 19) and U-100766 (Figure 19) are bactericidal for *S. pneumoniae* and bacteriostatic for most staphylococci and enterococci.

### 5.1. Combination Therapy with Other Antibiotics [21]

- Antagonism between Dup-721 and ciprofloxacin or norfloxacin was reported for MRSA, *S. epidermidis *and *E. faecalis. *
- In *In vivo* combination therapy studies, both drugs were found additive with vancomycin, gentamycin, or rifampin against *S. aureus*. Similarly, in a mixed *S. aureus *and *E. coli *infection model combination therapy with aztreonam or gentamycin as well as vancomycin, was found to be effective.
- Oxazolidinones are a new class of totally synthetic antimicrobial agents against multidrug-resistant gram-positive bacteria, including methicillin-resistant *Staphylococcus aureus* (MRSA), *Staphylococcus epidermidis* (MRSE), penicillin-resistant *Streptococcus pneunomiae* (PRSP) and vancomycin-resistant enterococci (VRE) [22–25]. Oxazolidinone has:
  - a novel mechanism of action,
  - selectively binding,
  - uniquely binding to the 50S ribosomal subunit,
  - inhibiting bacterial translation at the initiation phase of protein synthesis,
  - consequently, the drug would not show cross resistance with existing antibacterial agents.

The unique mechanism of action of oxazolidinones has attracted interest to develop derivatives with potent activity and broad spectrum. 

## 6. Structure Activity Relationship (SAR) [26, 27]

To find out the most potent and comparatively less toxic compounds, we need to to do exhaustive study of SAR.

**Figure 20:**
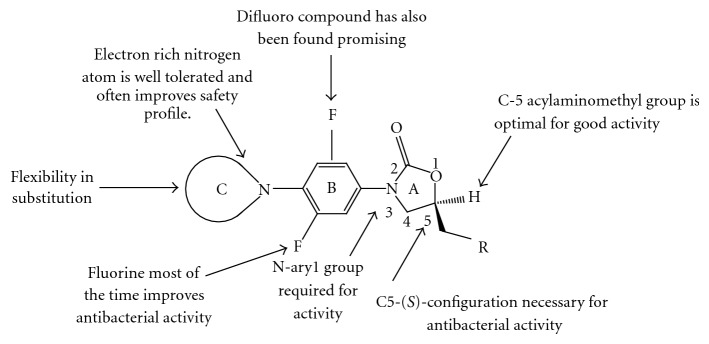


This paper cites the modifications directed for the different parts of the oxazolidinone template as shown in Figure 20. The “A” part manifests oxazolidinone ring, which bears aryl system on the 3rd position of the oxazolidinone ring termed as B region and the 4th position of the aryl group is extended by amine functionality that has been termed as C region. Oxazolidinone ring exclusively possess C5 side chain in the (*S*)-configuration, which has been optimized for the better efficacy.

### 6.1. Synthesized Derivative of Oxazolidinone by Modification

**Figure 21:**
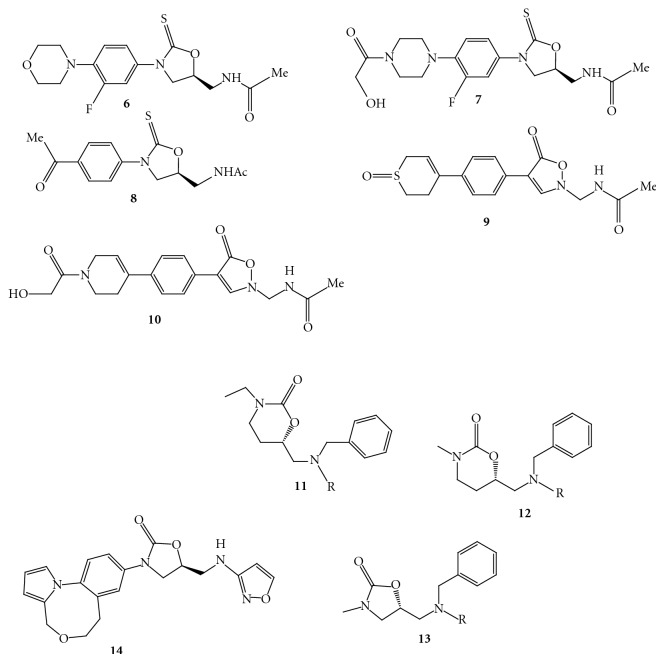


(1) *Modifications in the Region A with C5 *

- The thioanalogs of linezolid and eperezolid,** 6 **(Figure 21) and **7 **(Figure 21), do not seem to bind to the 50S ribosomal subunit of the bacteria and thereby are unable to inhibit bacterial protein synthesis. The molecular modeling study supported this unexpected finding of oxazolidine-2-thione [16]. The similar trend of antibacterial activity has been reported in the early investigation for compound **8 **(Figure 21), a thioanalogue of DuP 721 [28, 29].
- Quesnelle et al. have reported isoxazolidinone agents having various substitutions at the C-region and most of the compounds have been found to be potent against gram-positive strains [30]. The compounds **9 **(Figure 21) and **10 **(Figure 21) have shown broad-spectrum antibacterial activity, additionally the sulfoxide **9** exhibited less toxicity compared to the linezolid in a seven-day study in rats.
- Wang et al. have reported series of compounds with scaffolds **11**, **12**, and **13 **(Figure 21) containing chiral 1, 3 oxazinan-2-ones and oxazolidinones as basic core structures having tertiary amines core containing two aryl substituents [31].

(2)* Modifications in the Region B and at the C5 *[32]. Bosch et al. have reported convenient synthesis of conformationally constrained analog of linezolid **14 **(Figure 21) having a tricyclic moiety; however, no antibacterial activity has been disclosed for this compound.

**Figure 22:**
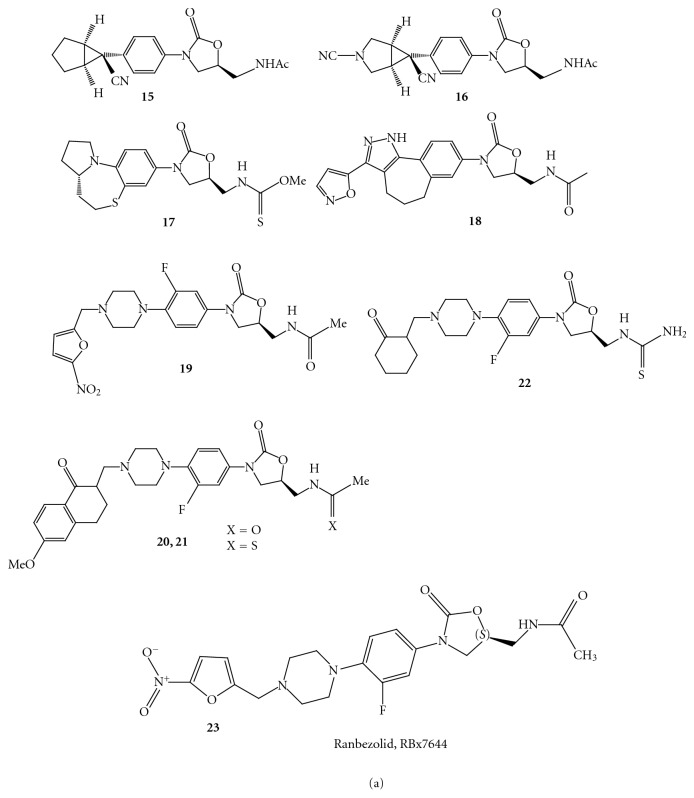


Merck has described novel oxazolidinone derivatives **15 **and **16 **substituted with cyclopropyl moiety [33]. The compounds** 15 (** Figure 22) and **16 **(Figure 22) exhibited impressive *in vitro *antibacterial activities against different strains** 15**.

Selvakumar et al. have reported constrained analogues of linezolid such as hexahydroazolo-quinoxaline and tetrahydroazolo-benzothiazine compounds [34]. Amongst the compound of this class the tetrahydroazolo-benzothiazine and thiocarbamate at C5 side chain **17 (** Figure 22) have shown antibacterial activity in the range of 0.25–1 *μ*g/mL against resistant and sensitive gram-positive strains. 

Choy et al. from Pfizer reported the synthesis of conformationally restricted oxazolidinone compounds [35]. The compound **18 **(Figure 22) was found to be more potent and exhibited broad-spectrum antibacterial activity with MIC values in the range of <0.06–0.25 *μ*g/mL for gram-positive organisms and 1-2 *μ*g/mL for fastidious gram-negative organisms.

(3) *Modifications in the Region C with C5 *[36]. A series of *N*-phenyl piperazinyl derivatives of oxazolidinone **19** (Figure 22) in which the nitrogen atom at 4-position of piperazinyl ring is substituted by different cinnamoyl groups. This optimization resulted in few potent compounds, which were found to be active against several gram-positive pathogens. In this class of compound some substituents were well tolerated on the phenyl ring of cinnamoyl group.

There is a reported synthesis of a few Mannich ketones of piperazinyl phenyl oxazolidinone derivatives and their antibacterial activity in various Gram-positive organisms such as *Bacillus subtilis*, *Staphylococcus aureus*, *Staphylococcus epidermidis, *and *Enterococcus faecalis* [37]. A moderately active compound **20 **(Figure 22) has been transformed to active compound **21 **(Figure 22). The compound **22 **(Figure 22) having cyclohexanone has shown antibacterial activities against various gram-positive strains with MIC values in the range of 1–4 *μ*g/mL, which is comparable to that of linezolid (0.5–4 *μ*g/mL).

**Figure 23:**
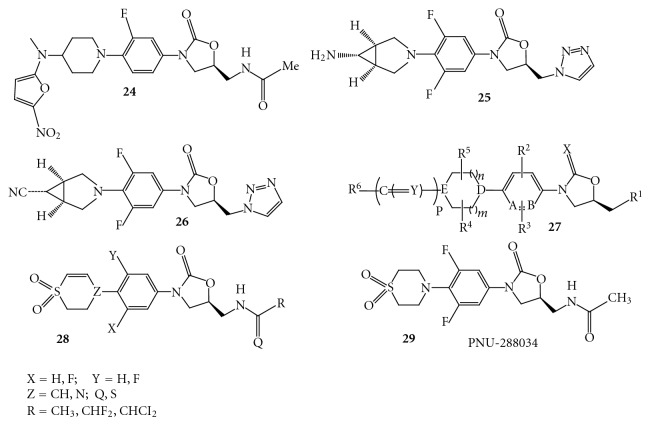


Ranbaxy has reported synthesis of oxazolidinones modified at the C region. The optimization of the series afforded a potent compound **23 **(Figure 22) (Ranbezolid, RBx7644), which is under clinical development [38]. They have further modified the compound **23 **to get the compound 2**4 **(Figure 23), wherein nitrofuran ring is attached to the 2° nitrogen of the aminopiperidine.

In another embodiment a group of scientists from Merck has published a patent describing novel oxazolidinone derivatives possessing cyclopropyl moiety [33]. The disclosed compounds **25 **and **26 **(Figure 23) have shown excellent *in vitro *antibacterial activity against broader panel of both susceptible and resistant strains.

Scientists from Orchid have described novel oxazolidinones **27 **(Figure 23) having variations in the regions B, C, and at the C5 side chain of oxazolidinone [39]. 

Pfizer has reported a series of oxazolidinone compounds **28 **(Figure 23) containing dihydrothiopyran substituent at the C region and instead of acetamide other variations such as halogenated amide and thioamide derivatives were studied at the C5 side chain [40]. The *in vitro *activities of the C5 modified derivatives were found to be superior compared to the corresponding C5 acetamide derivative. The compounds of this class have shown better antibacterial activities towards Gram-positive *Staphylococcus pneumoniae*, *Enterococcus faecium, *and Gram-negative *Haemophilus influenzae* as compared to the compound **29 (** Figure 23) (PNU-288034).

**Figure 24:**
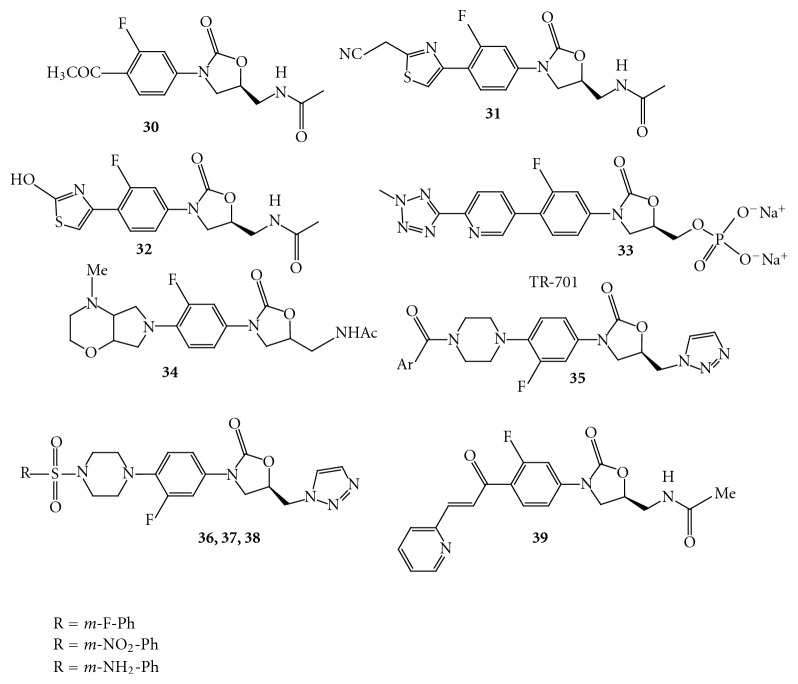


Zhai et al. have reported novel oxazolidinone derivatives having modified C-5 side chain. The compounds **30**, **31**, and **32 **(Figure 24) have shown inferior *in vitro *antibacterial activities in MIC assay against various strains [41]. 

Trius Therapeutics has announced the initiation of Phase 1 clinical trials of TR-701 **33 **(Figure 24) for the treatment of patients with serious Gram-positive bacterial infections, including those caused by methicillin-resistant *Staphylococcus aureus *and other drug-resistant strains [42]. 

A group of scientists from Ranbaxy has patented novel substituted phenyl oxazolidinone derivatives [43]. The compound **34 **(Figure 24) of this series has shown significant *in vitro *antibacterial activity in the MIC assay against different strains such as methicillin-resistant *Staphylococcus aureus *(4.0 *μ*g/mL; linezolid 2.0 *μ*g/mL), vancomycin-resistant *Enterococci* (2.0 *μ*g/mL; linezolid 2.0 *μ*g/mL). 

A series of arylcarbonyl and arylsulfonyl-piperizinyl-5-triazolylmethyl oxazolidinones **35 **(Figure 24) with improved activity against Gram-positive bacterial clinical isolates than Gram-negative bacterial clinical isolates [44]. 

Fan et al. have reported triazolyl oxazolidinones as antibacterial agents. Most of the analogues displayed activity superior to the linezolid and the vancomycin in various Gram-positive bacteria [45]. In the antibacterial MIC assay, the compounds **36, 37**, and **38 **(Figure 24) were found to be potent and further studied for their *in vivo *efficacies in mice model; however none of the compounds showed *in vivo *activity. 

**Figure 25:**
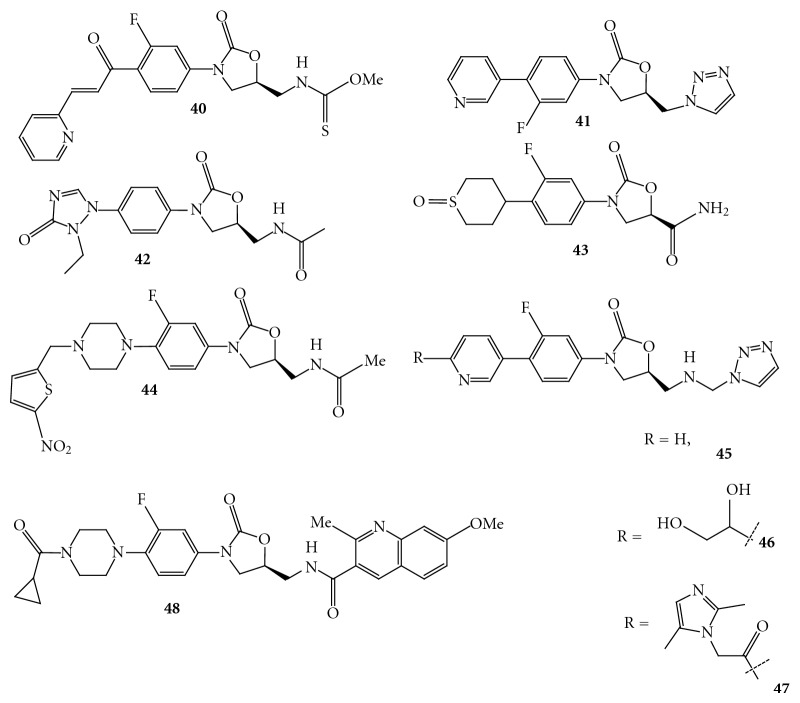


Selvakumar et al. have reported novel chalcone oxazolidinone hybrids with antibacterial activity. Of these, the compound **39 **(Figure 24) containing chalcone substituent in the C region showed significant antibacterial activities with MIC values of 4 *μ*g/mL against methicillin-resistant *Staphylococcus aureus *strain [46]. The acetamide group at C-5 was converted to thiocarbamate to get compound **40 **(Figure 25), which exhibited *in vitro *activity in the range of 0.25–2 ug/mL against resistant strains. 

Reck et al. at AstraZeneca have reported substituted (pyridin-3-yl) phenyloxazolidinones as antibacterial agents with reduced activity against monoamine oxidase A [47]. The compound **41** (Figure 25) showed excellent activity against Gram-positive bacteria; however the compound **41 **lacks the monoamine oxidase A inhibition and inhibits cytochrome P450 (CYP) due to its poor solubility.

Liu et al. have reported novel substituted oxotriazolylphenyl derivatives. Of the many compounds described, the compound **42 **(Figure 25) has shown potent antibacterial activity against pathogens *Staphylococcus epidermidis*, *Streptococcus pneumoniae*, *Streptococcus enteritidis, and Streptococcus nonhemolyticus *at 0.10–6.25 *μ*g/mL [48] O Vara Prasad et al. have reported new series of oxazolidinones with C5 carboxamide functionality, that is, reverse amides, which blocks the bacterial protein synthesis. These compounds have also exhibited less potency against monoamine oxidase enzymes, indicative for the lower side effects. The compound **43 **(Figure 25) has shown reduction of myelotoxicity in a 14-day safety study in rodents and compared with linezolid [49]. 

Rudra et al. have reported series of heterocyclic oxazolidinones as antibacterial agents with identification of RBx 8700 as potent compound [50]. Systematic modification of the linker between the five-membered heterocycle and the piperazinyl ring of RBx 7644 and its thienyl analogue **44 **(Figure 25) resulted in the identification of an expanded spectrum compound. 

Reck et al. reported novel acyclic substituted (pyridin-3-yl) phenyl oxazolidinones **45**–**47 **[51] (Figure 25). The acyclic 6-substituted pyridin-3-yl phenyl oxazolidinones **46 **and **47 **having bulky substituent demonstrated excellent antibacterial activity against Gram-positive organisms, including linezolid resistant *Streptococcus pneumonia *(1.0 *μ*g/mL). The compounds with bulkier substituents showed reduced MAO-A activity (Ki = 40 *μ*M; Ki = 19 *μ*M) compared to the corresponding unsubstituted parent compound **48** (Figure 25) (Ki < 0.3 *μ*M) and the improved solubility. 

The high-molecular-weight novel oxazolidinone derivatives having variations in the regions B, C, and at the C5 side chain have been patented by a group of scientists from Ferrer International Company [52].

**Figure 26:**
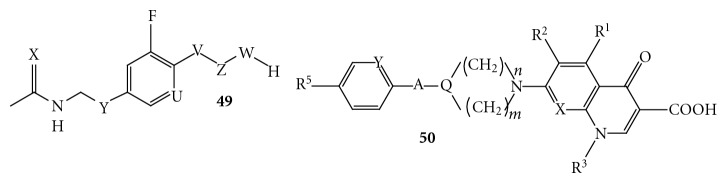


(4) *Hybrid Molecules *[53]. Morphochem AG has published a patent mentioning oxazolidinone-quinolone hybrid **49 **(Figure 26). However, no discloser has been made for their antibacterial activities. Further, in another report they have described different variant of oxazolidinone-quinolone hybrids of type **50**. The disclosed derivatives **50 **(Figure 26) possess the pharmacophore of quinolone and oxazolidinone linked together through a linker and the hybrid has been used as an antibacterial agent. However, there is no mention of the antibacterial activities for the disclosed oxazolidinone-quinolone hybrids.

(5) *Computational Studies. *Extensive studies on the syntheses of oxazolidinone antibacterial agents and their SAR have been reported; however only few QSAR studies have been published. The first QSAR study was reported in the year 1999, comprised a 3D-QSAR study on a small data sets of two novel series of oxazolidinone antibacterial agents using “Comparative Molecular Field Analysis” (CoMFA), wherein a training set of 17 compounds with two reference compounds have been used. The CoMFA steric, electrostatic fields, and ClogP were used as descriptors, and the activities against methicillinresistant *Staphylococcus aureus *88 (MRSA 88) as dependents were developed. The cross-validated r2cv (0.653) and conventional r2 (0.984) from the Partial Least Square (PLS) and CoMFA analyses indicated considerable reliability for predicting the antibacterial activities of oxazolidinone antibacterial agents. 

The second 3D-QSAR studies, reported by Karki and Kulkarni in the year 2001, used a genetic function algorithm with a data set of 60 compounds. The QSAR models were developed using a training set of 50 compounds and the *in vitro *MIC against *Staphylococcus aureus *SFCO-1. The r2 values reported for the models range from 0.629 to 0.732. The predictive ability of the QSAR model was evaluated with a test set of 10 compounds. The results obtained concluded that the antibacterial activity of the 3-aryloxazolidin-2-ones is strongly dependent on the electronic factor as expressed by lowest unoccupied molecular orbital energy (LUMO) and the spatial factor as expressed by density and thermodynamic factors accounted for molar refractivity and the heat of formation. The SAR study by Tokuyama et al. revealed that the antibacterial activity against Gram-positive bacteria including methicillin-resistant *Staphylococcus aureus *and vancomycin-resistant enterococci on 5 thiocarbonyl oxazolidinones was significantly affected by the lipophilicity, especially the calculated log *P* value, the balance between 5-hydrophilic (or hydrophobic) substituents and hydrophobic (or hydrophilic) substituents on the benzene ring (Table 2).

### 6.2. Oxazolidinone in Clinical Trials

#### 6.2.1. Oxazolidinone: Biological Activity

The development of resistance by the antibiotics in the Gram-positive pathogenic bacteria over the last twenty years and continuing today has created a need for new antibiotic classes, which may be unaffected by existing bacterial resistance. The oxazolidin-2-ones not only represent a new class with a novel mechanism of action, but also satisfy the requirement for overcoming the resistance mechanisms. Both linezolid and eperezolid, the first chemical candidates, arose from the piperazine subclass, with the first one being chosen further development because of its enhanced pharmacokinetic properties. The main attractive traits of the oxazolidinone series have encouraged further work in the area, and the patent literature reveals that extensive chemical investigation is currently being made. The unexpected early resistance development emphasizes the need for further exploration of features of the oxazolidinone to eliminate these deficiencies. Recently, several changes, involving the C5 side chain as well the N-phenyl heterocyclic ring, give promise for such improvement. Various biological activity show by oxazolidinone derivatives like the following.

 (1)* Antibacterial Activity* [54]. Linezolid is an oxazolidinone developed by Pharmacia (formerly Pharmacia & Upjohn) for the treatment of multiresistant Gram-positive infections 187765, 317456. Linezolid resistance due to a 23S rRNA mutation may emerge in Enterococci during therapy with this antimicrobial and may be associated with clinical failure 368652. Following FDA approval, linezolid was launched in May 2000 368526, 368652. In April 2000, the FDA approved linezolid injection, tablets, and oral suspension for the treatment of patients with infections caused by Gram-positive bacteria. It is indicated for adults in the treatment of nosocomial pneumonia, community-acquired pneumonia (CAP), complicated and uncomplicated skin and skin structure infections and vancomycin-resistant enterococcus (VRE) infections caused by methicillin-resistant *Staphylococcus aureus* (MRSA), VRE, faecium and penicillin-susceptible *Streptococcus pneumoniae* 363503.

Vancomycin resistance was reported in only 0.6 and 3.0% of *Enterococcus faecalis* and *E. faecium*, respectively. Penicillin resistance occurred in 25.1% of *Streptococcus pneumonia*, 4.9% at the high level (≥2 mg/L). The MIC_90_ for linezolid was 1 mg/L for streptococci and 2 mg/L for enterococci and staphylococci. Using the US FDA- and EUCAST-recommended susceptible breakpoints for linezolid, there were no confirmed reports of linezolid resistance minimum inhibitory concentration (MIC), ≥8 mg/L. The distribution of linezolid MIC values was unimodal and varied between 0.25 and 1 mg/L for streptococci (>90% of isolates) and between 1 and 2 mg/L for staphylococci (>90%) and enterococci (>95%).

**Figure 27:**
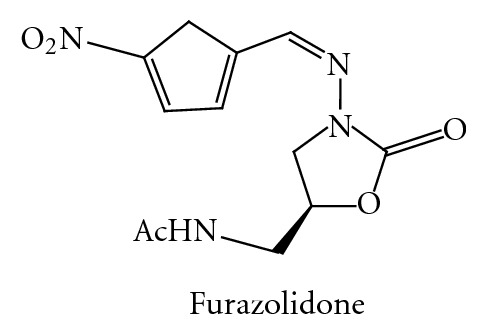


Favorable clinical results shown by linezolid prompted many pharmaceutical industries and academic institutions to explore the possibilities of expansion of antibacterial spectrum of this class. A large number of publications, reviews, and patents testify to the interest of the various research groups in the oxazolidinone class of antibacterials. Furazolidone as shown in Figure 27 is the first member of the oxazolidinone class discovered in 1950 and appears to be the initial candidate responsible for the genesis of further work on oxazolidinone antibacterials.

**Figure 28:**
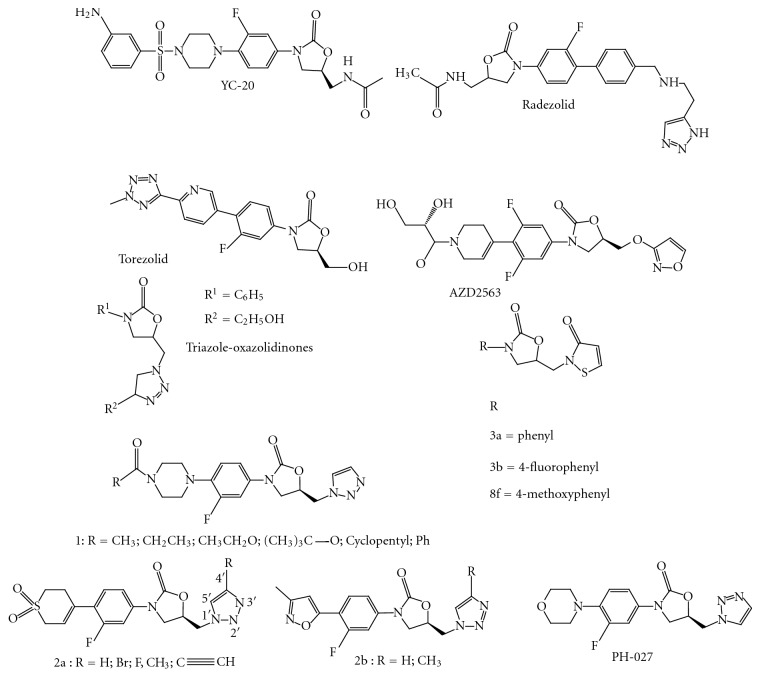


YC-20 [55] (Figure 28) and linezolid were active against all Gram-positive organisms isolated, including strains resistant to other classes of antibiotics. YC-20 exhibited MIC50 and MIC90 values of <0.5 and 2 mg/L against all isolates. Overall, the activity of YC-20 is slightly superior to that of linezolid. The present study confirms and expands previous findings of the good *in vitro* activity of YC-20 against Gram-positive organisms. YC-20 has a potential role in the treatment of infections caused by Gram-positive pathogens, especially for multidrug-resistant Gram-positive bacteria (Table 3). 

Radezolid (RX-1741) [56, 57] (Figure 28) is a novel oxazolidinone with broader spectrum of coverage and increased activity against Gram-positive organisms as compared to other oxazolidinones. Radezolid has recently completed successfully two Phase 2 clinical trials: one for community-acquired pneumonia (CAP) and the second for uncomplicated skin and skin structure infections (Usssi) (Table 4).

The spectrum of activity of torezolid (TR-700) (Figure 28), the active moiety of torezolid phosphate (TR-701), and proposes tentative MIC and disk diffusion breakpoints as well as quality control ranges [58]. The *in vitro* susceptibilities of 1,096 bacterial isolates, representing 23 different species or phenotypic groups, were determined for torezolid, linezolid, cefotaxime, and levofloxacin using Clinical and Laboratory Standards Institute (CLSI) broth microdilution MICs, minimum bactericidal concentrations (MBCs), agar dilution, and disk diffusion testing methods. Torezolid was very active against the majority of Gram-positive strains, including methicillin-susceptible and -resistant *Staphylococcus aureus* (MIC_50_ = 0.25 *μ*g/mL, MIC_90 _≤0.5 *μ*g/mL), coagulase-negative staphylococci (CNS; MIC_50_ = 0.25 *μ*g/mL, MIC_90 _≤0.5 *μ*g/mL), enterococci (MIC_50_ and MIC_90 _≤0.5 *μ*g/mL), and streptococci (MIC_50_ and MIC_90 _≤0.25 *μ*g/mL). Based upon MIC_90 _s, torezolid was 4-fold more active than linezolid against *S. aureus*, coagulase-negative staphylococci, and the enterococci and 8-fold more active than linezolid.

In 2002 AstraZeneca introduced posizolid (AZD2563) (Figure 28). Results indicate that posizolid has excellent, targeted bactericidal activity against all common gram-positive bacteria, regardless of resistance to other classes of antibiotics [59].

The *in vitro* activity of AZD2563, a novel oxazolidinone, was assessed against 595 Gram-positive cocci, comprising recent surveillance isolates and a collection of resistant (including multiresistant), epidemiologically diverse isolates [60]. The MICs of AZD2563 for staphylococci, pneumococci, and enterococci had narrow ranges, 0.25–2 mg/L, with modal MICs of 1 mg/L for staphylococci and pneumococci, and 1–2 mg/L for enterococci. AZD2563 was equally active against the surveillance isolates and those that had been selected for their multiresistance to other agents. The MICs of AZD2563 were either the same as those of linezolid or twofold lower.

A few Mannich ketones of piperazinyl oxazolidinone derivatives have been synthesized and their antibacterial activities in various Gram-positive organisms such as *Bacillus subtilis, Staphylococcus aureus, Staphylococcus epidermidis, *and *Enterococcus faecalis *were evaluated by MIC determination [61]. The analog showed inferior activity than linezolid as well as eperezolid. Thus in an attempt to improve potency, we prepared several analogues by modifying the ketone part A. However, all such resulting compounds lost their *in-vitro* antibacterial activity. It has been reported that thioacetamide at the 5th position of the oxazolidinone improves the activity. MIC was determined by microbroth dilution technique and values reported in table represent the highest MIC value obtained in triplicate. S.a1, *Staphylococcus aureus *ZYABL 006; S.a2, *Staphylococcus aureus *ATCC 33591; S.e, *Staphylococcus epidermidis *ATCC 12228, B.s, *Bacillus subtilis *ATCC 6633; E.f, *Enterococcus faecalis *ATCC 29212.

The compound library (Figure 28) was screened using a disk diffusion assay on the Gram-positive bacterial reference strains, *M. smegmatis* ATCC 14468, *Bacillus subtilis* ATCC 6633, and *Enterococcus faecalis* ATCC [62]. *Mycobacterium smegmatis* was inoculated into Middlebrook 7H9 containing 0.2% glycerol and ADC enrichment while the other strains were inoculated into Mueller–Hinton broth. The inocula of all strains were allowed to incubate for approximately 24 h at 37°C and 180 rpm. Minimum inhibitory concentrations (MICs) of compound was also determined against the same strains using a broth macrodilution assay using either Middlebrook 7H9 containing 0.2% glycerol and ADC enrichment or Mueller–Hinton broth 20. The highest concentration for each compound tested was 32 lg/mL, and each subsequent tube was a twofold dilution of the previous. The M. smegmatis tubes were incubated for 48 h at 37°C while the other strains were incubated for 24 h and then analyzed. Thiazolyl blue tetrazolium bromide (MTT) was dissolved in MeOH (10 mg/mL) and added to the solution (20 lL) to aid in bacteria visualization.

The *in vitro* antibacterial activities of the compound and vancomycin, ciprofloxacin, and linezolid as the reference drugs were determined by the conventional agar dilution method using Mueller–Hinton agar medium [63]. The tested Gram-positive organisms included two clinical isolates of *S. aureus* resistant to methicillin (MRSA), *S. aureus* ATCC 29737, and *S. epidermidis* ATCC 12229. Gram-negative bacteria used in the study were *E. coli* ATCC 8739,* S. typhimurium* ATCC 1639 and *P. aeruginosa* ATCC 9027. The tested compounds, vancomycin and ciprofloxacin, were dissolved in DMSO while linezolid was dissolved in water. Suspensions of each of bacteria were prepared to contain approximately 106 colony-forming units (CFU/mL) and applied to plates with twofold serially diluted compounds to be tested in distilled water in concentration ranging from 0.01 to 100 mg/mL and incubated at 37°C for 18 h. To ensure that the solvent had no effect on bacterial growth, a control test was performed with test medium supplemented with DMSO at the same dilution as used in the experiments.

Minimum inhibitory concentrations (MICs, mg/mL) were determined on Mueller–Hinton (MH) agar with medium containing dilutions of antibacterial agents ranging from 0.12 to 64 mg/mL [64]. The test compounds were dissolved in 20% water in DMSO, while linezolid and vancomycin were dissolved in 40% water in ethanol and water, respectively. The tests were performed using MH agar plates for all staphylococci and enterococci and on MH agar plates supplemented with 5% sheep blood to facilitate the growth of *S. pneumoniae*, *H. influenzae*, and *M. catarrhalis*. The Gram-positive clinical isolates utilized in this study consisted of methicillin-resistant *S. aureus* (MRSA, *n* = 1/4  10), methicillin-susceptible S. aureus (MSSA, *n* = 1/4 10), methicillin-resistant coagulase-negative staphylococci (MR-CNS, *n* = 1/4 3), methicillin-sensitive coagulase-negative staphylococci (MS-CNS, *n* = 1/4 6), S. pneumoniae (*n* = 1/4  3), vancomycin-sensitive (VSE, *n* = 1/4  6) and vancomycin-resistant (VRE,  *n* = 1/4  4) enterococci. The Gram-negative clinical isolates tested included *H. influenzae* (*n* = 1/4  4) and *M. catarrhalis* (*n* = 1/4  1). The reference strains, *S. aureus* ATCC 25923, *S. epidermidis* ATCC 12228 and *E. faecalis* ATCC 29212, *E. coli* ATCC 25922, and *H. influenzae* ATCC 49247 were used as controls. The final bacterial concentration for inocula was 107 CFU/mL and was incubated at 35°C for 18 h. The test compounds were also evaluated against *S. aureus* ATTC 25923 in MH broth supplemented with 50% human plasma to assess the extent of plasma binding and/or plasma instability. The MIC was defined as the lowest drug concentration that completely inhibited growth of the bacteria.

Against some of the gram-positive bacteria strains tested, the triazole oxazolidinone (Figure 28) PH-027 demonstrated MIC values comparable to or 1- to 2-fold lower that hose of linezolid and vancomycin [65]. In particular, the MIC values of PH-027 against vancomycin susceptible *E. faecium* (VSE), vancomycin-intermediate-resistant *E. faecalis* (VIRE), and vancomycin-resistant E. faecalis (VRE) were 0.5, 0.5, and 2 mg/mL, respectively, compared to an MIC of 2 mg/mL demonstrated by linezolid against these strains. However, the MIC's of vancomycin against the same strains were 4, 8 and >32 mg/mL, respectively. PH-027 demonstrated the most potent antibacterial activity against both sensitive and resistant gram-positive bacteria strains, including MRSA, MSSA, MS-CNS, MR-CNS, PRSP, and enterococci (*Enterococcus faecium* and *Enterococcus faecalis*). 

**Figure 29:**
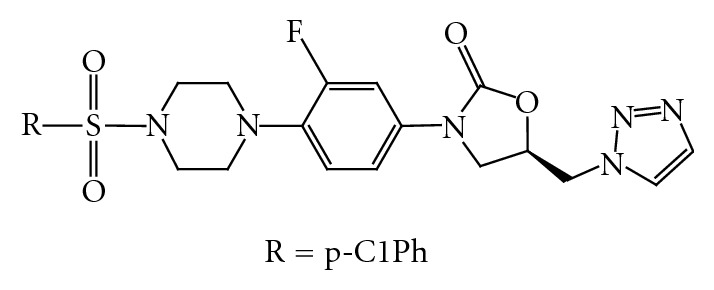


**Figure 30:**
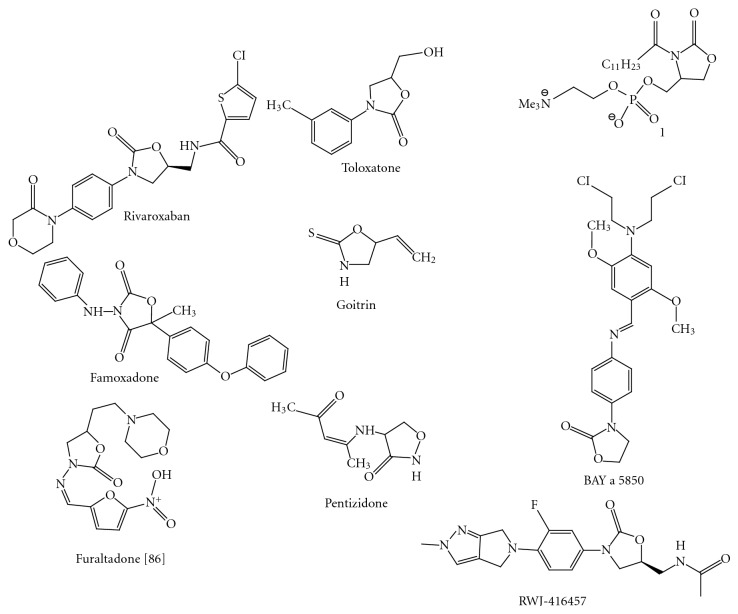


The result of *in vitro* antibacterial activity against a spectrum of resistant and susceptible Gram-positive organisms [66] clearly shows that all compounds (Figure 29) bearing sulfonyl group have good antibacterial activity. Compound showed more potent antibacterial activity than linezolid and vancomycin. Obviously, the introduction of strong electron-withdrawing groups (e.g., CF3, F, and NO2) into the oxazolidinones can confer excellent antibacterial activity especially at the 3rd position of the phenylsulfonyl group, in addition, electron-donating groups (e.g., Cl).

(2)* Anticoagulant Activity* [67, 68]. Rivaroxaban (BAY 59–7939) (Figure 30) is an oral anticoagulant invented and manufactured by Bayer; in a number of countries it is marketed as Xarelto. If approved by the United States FDA, it will be marketed by Ortho-McNeil Pharmaceutical. It is the first available orally active direct factor Xa inhibitor. Rivaroxaban is well absorbed from the gut and maximum inhibition of factor Xa occurs four hours after a dose. The effect lasts 8–12 hours, but factor Xa activity does not return to normal within 24 hours so once daily dosing is possible. 

 (3)* Antitubercular Activity. *During the course of investigation in the oxazolidinones antibacterial agent area was identified a subclass with especially potent in vitro activity against mycobacteria [69]. The salient structural feature of these oxazolidinone analogues, U-100480, U-101603, and U-101244, is their appended thiomorpholine. Potent activity against a screening strain of *M. tuberculosis* was determined by U-100480 and U-101603 (MIC = 0.125 mg/mL).

The activities of linezolid, eperezolid, and PNU-100480 were evaluated in a murine model of tuberculosis. Approximately 10 (7) viable *Mycobacterium tuberculosis* ATCC 35801 organisms were given intravenously to 4-week-old outbred CD-1 mice [70]. In the first study, treatment was started 1 day postinfection and was given by gavage for 4 weeks. Viable cell counts were determined from homogenates of spleens and lungs. PNU-100480 was as active as isoniazid. Linezolid was somewhat less active than PNU-100480 and isoniazid. Eperezolid had little activity in this model. In the next two studies, treatment was started 1 week postinfection. A dose-response study was performed with PNU-100480 and linezolid (both at 25, 50, and 100 mg/kg of body weight). PNU-100480 was more active than linezolid, and its efficacy increased with an escalation of the dose. Subsequently, the activity of PNU-100480 alone and in combination with rifampin or isoniazid was evaluated and was compared to that of isoniazid-rifampin. The activity of PNU-100480 was similar to that of isoniazid and/or rifampin in the various combinations tested. Further evaluation of these oxazolidinones in the murine test system would be useful prior to the development of clinical studies with humans.

 (4) *Antidepressant or Psychotropic Activity. *Monoamine oxidase (MAO) inhibitors were developed as antidepressants but many drugs, including the novel oxazolidinone antibacterial agents, share similar molecular properties and have MAO inhibitory activity [71]. Factors important for binding antidepressants and modifications to decrease binding of oxazolidinones to avoid undesirable vascular effects are discussed [72].

 In man, the antidepressant agent 3-(3-methylphenyl)-5-hydroxymethyl-2-oxazolidinone (toloxatone) (Figure 30) on oral dosing was mainly eliminated in urine (80% dose in 12 h).

Plasma concnentration of total radioactivity was max (5.8 *μ*g equiv./mL) at 30 min to 1 h after administration and declined rapidly (*t*_1/2_, 1.25 h). Unchanged drug accounted for 48, 32, and 13% of plasma radioactivity at 15 min, 1 h, and 6 h, respectively.

The drug was extensively metabolized. The major urinary metabolites were 3-(3-carboxyphenyl)-5-hydroxymethyl-2-oxazolidinone and a glucuronide of toloxatone. A minor urinary metabolite, characterized as a phenolic derivative, was also excreted conjugated. 

On the basis of previous laboratory studies AS-8 (5-morpholinemethyl-3-(4-chlorobenzylideneamino)-2-oxazolidinone) was suggested to possess antidepressant-like activity. Forced swim test, learned helplessness, and conflict Vogel's test were performed after three prior administrations of AS-8 (24, 5, and 1 h before the test). The data have shown that AS-8 produces moderate antidepressant effect but did not induce anxiolytic-like action. Biochemical data revealed increased brain 5-HT and 5-HIAA levels following AS-8 administration [73]. The combined treatment of rats with AS-8 (100 mg/kg) and amitriptyline (5 mg/kg) or desipramine (1.25 mg/kg) significantly stimulated active behavior in the forced swim test above the level obtained with each of the drug given separately. The present data suggest the potential antidepressant efficacy of AS-8 in conjunction with small doses of tricyclic antidepressants.

 (5) *Phospholipase Inhibitor* [74]. (S)- and (R)-3-dodecanoyl-4-phosphatidylcholinohydroxymethyl-2-oxazolidinone **(1) **(Figure 30), which are cyclic analogues of the amide phospholipid 7, were synthesized. The inhibitory activities of these analogues toward phospholipase A_2_ were compared with that of the amide analogue 7. (S)- and (R)-3-dodecanoyl-4-phosphatidylcholinohydroxymethyl-2-oxazolidinone (1) were synthesized. The inhibitory activities of these analogues toward phospholipase A_2_ were compared with that of the amide analogue.

 (6) *Agriculture Fungicide* [75]. 5-Methyl-5-(4-phenoxyphenyl)-3-phenylamino-2,4-oxazolidinedione, DPX-JE874, is a new agricultural fungicide under development by DuPont. DPX-JE874 is a member of a new class of oxazolidinone fungicides which demonstrate excellent control of plant pathogens in the Ascomycete, Basidiomycete, and Oomycete classes which infect grapes, cereals, tomatoes, potatoes, and other crops. The synthesis, mode of action, and structure-activity relationships of two types of oxazolidinones, 2-thioxo-4-oxazolidinones and 2,4-oxazolidinediones, were discussed.

Famoxadone (3-anilino-5-methyl-5-(4-phenoxyphenyl)-1, 3-oxazolidine-2, 4-dione) (Figure 30), is a new agricultural fungicide recently commercialized by DuPont under the trade name Famoxate [76]. Famoxadone is a member of a new class of oxazolidinone fungicides that demonstrate excellent control of plant pathogens in the Ascomycete, Basidiomycete, and Oomycete classes that infect grapes, cereals, tomatoes, potatoes, and other crops.

Famoxadone is a preventative and curative fungicide recently commercialized for plant-disease control [77]. The molecule and its oxazolidinone analogs are potent inhibitors of mitochondrial ubiquinol: cytochrome c oxidoreductase (cytochrome bc_1_) and they bind in the Q_0_ site of the enzyme near the low potential heme of cytochrome b. Inhibitor binding constants for five mutant cytochrome bc_1_ enzymes from *Saccharomyces cerevisiae* having single amino acid changes in their apocytochrome b located near the low potential heme were compared with their two parental wild-type enzymes. The five individual amino acid changes altered the inhibition constants for the inhibitors famoxadone, myxothiazol, azoxystrobin, and kresoxim-methyl in dissimilar fashion.

 (7) *CNS Depressant* [78, 79]. A method of treating central nervous system diseases and disorders, the diseases and disorders being responsive to drugs possessing psychotropic activity. The method involves administering to a subject suffering from the disease or disorder a therapeutically effective amount of 5-morpholinomethyl-3-(4-chlorobenzylideneamine)-2-oxazolidinone or a pharmaceutically acceptable salt of 5-morpholinomethyl-3-(4-chlorobenzylideneamine)-2-oxazolidinone. The invention also relates to a pharmaceutical composition, the composition having pharmaceutically acceptable carriers and 5-morpholinomethyl-3-(4-chlorobenzylideneamine)-2-oxazolidinone and/or a pharmaceutically acceptable salt of 5-morpholinomethyl-3-(4-chlorobenzylideneamine)-2-oxazolidinone.

 (8) *Centrally Acting Muscle Relaxants* [80]. The severity of anaemic decerebrate rigidity was quantitatively determined by measuring the frequency of electromyographic potentials in the rat. Some oxazolidinones markedly reduced the severity of this decerebrate rigidity in a dose-dependent manner, (4S,5R)-4-(2-methylpropyl)-3-[3-(perhydroazepin-1-yl)propyl]-5-phenyl-1,3-oxazolidin-2-one (MLV-6976) being the most potent. In addition to the oxazolidinones, an amino alcohol derivative, (1RS,2SR)-5-methyl-1-phenyl-2-(3-piperidinopropylamino)hexan-1-ol(MLV-5860) also reduced the rat decerebrate rigidity. In the oxazolidinone series, the optical isomers with absolute configuration (S) at the 4-position were more potent than the corresponding (4R)-isomers, while there was no significant difference in their LD50 values. Normal rats and mice receiving MLV-6976 at doses which reduced decerebrate rigidity showed no behavioural changes, impairment of motor coordination only appearing at extremely high doses. MLV-6976 and its derivatives did not affect spinal reflex potentials in cats. MLV-6976 reduced the severity of harmaline-induced tremor in mice in a dose-dependent manner, but slightly augmented tremorine-induced tremor. The frequency of the spike discharges induced by iontophoretically applied glutamate was reduced by MLV-6976 in a dose-dependent manner in rat cortical neurones. The amplitude of miniature endplate potentials of the rat diaphragm was decreased by MLV-6976 only at concentrations greater than 0.1 mM. It is concluded that MLV-6976 acts on the brainstem or/and higher levels of the brain rather than on the spinal cord or the peripheral nervous system to reduce the excessive activities of the nervous system.

 (9) *Antithyroid Agent* [81]. 5-Vinyloxazolidine-2-thione (VOT) administered orally to lactating rats was found to be efficiently transferred to the sucklings via the milk. In mothers the exposure to VOT resulted in an increased percentage of neutrophils, a decreased percentage of lymphocytes, and increases in the relative weights of liver and thyroid. Suckling rats showed a decreased number of leucocytes, increases in the relative weights of liver and thyroid and structural changes in the thyroid. Male sucklings were more affected than female pups. The antithyroid effects were clearly related to the maternally administered VOT doses.

RS-Goitrin (Figure 30) can be conveniently prepared by a simplification of the Ettlinger procedure. Goitrin is a moderate inhibitor of purified bovine adrenal dopamine beta-hydroxylase [82]. The administration of goitrin leads to a depression of brain norepinephrine and to an elevation of heart and adrenal dopamine.

Brassica vegetables are the major source of glucosinolates in the human diet. Certain glucosinolates are readily converted into goitrogenic species, notably 5-vinyloxazolidine-2-thione and thiocyanate ion [83]. The effect of dietary Brussels sprouts, a particularly rich source of such glucosinolates, on thyroid function has been examined.

 (10) *Antiblastic Activity in Chemotherapy *[84]. Antiblastic activity means retardation of growth. 3-p-(2′, 5′-Dimethoxy-4′-(N,N-bis-(-chloroethyl)-amino)benzylideneamino)phenyl-2-oxazolidinone (GEA 29; BAY a 5850) (Figure 30) and its analogues use as antiblastic agent.

Some other oxazolidinone derivatives are used as anticancer in early clinical trial for example,

- 3-nitroso-5-methyl-2-oxazolidone,
- 3-(2-hydroxy-3-(2-nitro-1H-imidazol-1-yl)propyl)-2-oxazolidinone,
- 4-(4-(bis(2-chloroethyl)amino)-2,5-dimethoxyphenyl) methylene aminophenyl)-2-oxazolidinone.

(11) *Use in Urinary Tract Infection* [85]. The antimicrobial activity of linezolid, a recently developed antibiotic agent active against Gram-positive bacteria, was tested against pathogens from three different collections. (1) Uropathogens from hospitalized urological patients (1990/1991) with complicated and/or hospital-acquired UTIs; Urologic Clinic, Hospital St. Elisabeth, Straubing. (2) Uropathogens from a multicentre study (1995/1996) comprising 37 urological centres throughout Germany. (3) MRSA isolates of patients and staff (1999/2000) within the Hospital St. Elisabeth, Straubing. Genotyping of the latter isolates was performed by pulsed-field electrophoresis. The minimal inhibitory concentrations (MICs) of linezolid determined by an agar (Isosensitest) dilution method using a multipoint inoculator and an inoculum of 104 cfu per point ranged for methicillin susceptible *Staphylococcus aureus* (MSSA) (**n** = 27) between 2 and 4 mg/L, for methicillin resistant *S. aureus* (MRSA) (**n** = 35) between 1 and 2 mg/L, for methicillin susceptible coagulase-negative staphylococci (CNS) (MSSE) (**n** = 67) between 0.5 and 4 mg/L, for methicillin resistant CNS (MRSE) (**n** = 19) between 0.25 and 2 mg/L, for *Enterococcus. faecalis* (**n** = 184) between 0.5 and 4 mg/L, for *E. faecium* (**n** = 3) 2 mg/L, and for *Streptococcus* spp. (**n** = 4) between 0.25 and 1 mg/L, indicating that all strains were susceptible. According to the *in vitro* activity, linezolid may be considered a promising antibacterial agent for the treatment of complicated UTI caused by Gram-positive uropathogens. Thus, linezolid should be evaluated in a clinical study (Table 5).

 (12) *Cycloserine Analogues* [86]. (R)-4-[(1-Methyl-3-oxo-1-butenyl)amino]isoxazolidin-3-one is used as cycloserine analogues. Synonym of this compound is Pentizidone (Figure 30).

D-Cycloserine is a broad-spectrum antibiotic used with other antibiotics to treat various forms of tuberculosis [87]. Its prodrug sodium (R)-4-[(1-methyl-3-oxo-1-butenyl)amino]-3-isoxazolidinone hemihydrate, developed for better aqueous stability and solubility, is combined with another broad-spectrum antibiotic, fludalanine.

#### 6.2.2. Current Research Work on Oxazolidinone

Mathur et al. demonstrated RBx 11760 MICs were in the range of 0.5–1 mg/L for *C. difficile* isolates, and it demonstrated concentration-dependent killing of *C. difficile* ATCC 43255 and *C. difficile* 6387 up to 2–4x MIC (1–2 mg/L). RBx 11760, at concentrations as low as 0.25–0.5 mg/L, resulted in a significant reduction in *de novo* toxin production as well as sporulation in different *C. difficile* isolates [88]. In contrast, vancomycin, metronidazole, and linezolid had little or no effect on toxin production and appeared to promote the formation of spores. In the hamster infection model, treatment with RBx 11760 resulted in prolonged survival of animals as compared with vancomycin or metronidazole, which correlated well with the histopathology results. Macromolecular labelling results suggest that RBx 11760 is a potent inhibitor of bacterial protein synthesis. RBx 11760 showed excellent *in vitro* and *in vivo* activity against *C. difficile*, and it could be a promising novel candidate for future drug development against *C. difficile* infection.

 Skripkin et al. have reported new and improved antibiotics are urgently needed to combat the ever-increasing number of multidrug-resistant bacteria [89]. In this study, we characterized several members of a new oxazolidinone family, R*χ*-01. This antibiotic family is distinguished by having *in vitro* and *in vivo* activity against hospital-acquired, as well as community-acquired, pathogens. We compared the 50S ribosome binding affinity of this family to that of the only marketed oxazolidinone antibiotic, linezolid, using chloramphenicol and puromycin competition binding assays. The competition assays demonstrated that several members of the R*χ*-01 family displace, more effectively than linezolid, compounds known to bind to the ribosomal A site. We also monitored binding by assessing whether R*χ*-01 compounds protect U2585 (*Escherichia coli* numbering), a nucleotide that influences peptide bond formation and peptide release, from chemical modification by carbodiimide. The R*χ*-01 oxazolidinones were able to inhibit translation of ribosomes isolated from linezolid-resistant *Staphylococcus aureus* at submicromolar concentrations. This improved binding corresponds to greater antibacterial activity against linezolid-resistant enterococci. Consistent with their ribosomal A-site targeting and greater potency, the R*χ*-01 compounds promote nonsense suppression and frameshifting to a greater extent than linezolid. Importantly, the gain in potency does not impact prokaryotic specificity as, like linezolid, the members of the R*χ*-01 family show translation 50% inhibitory concentrations that are at least 100-fold higher for eukaryotic than for prokaryotic ribosomes.

Hilliard et al. have reported RWJ-416457 is an investigational pyrrolopyrazolyl-substituted oxazolidinone with activity against antibiotic-susceptible and -resistant gram-positive pathogens [90]. Efficacies of RWJ-416457, linezolid, and vancomycin against methicillin-susceptible *Staphylococcus aureus* (MSSA) and community-associated methicillin-resistant *S. aureus* (CA-MRSA) in murine skin and systemic infections were compared, as were efficacies against *Streptococcus pneumoniae* in a lower respiratory infection. In staphylococcal systemic infections, RWJ-416457 was equipotent to twofold more potent than linezolid, with 50% effective dose values ranging from 1.5 to 5 mg/kg of body weight/day. RWJ-416457 was two- to fourfold less potent than vancomycin against MSSA but up to fourfold more potent than vancomycin against CA-MRSA. In MSSA and CA-MRSA skin infections, RWJ-416457 demonstrated an efficacy similar to that of linezolid.

Locke et al. have reported that TR-700 (torezolid), the active moiety of the novel oxazolidinone phosphate prodrug TR-701, is highly potent against gram-positive pathogens, including strains resistant to linezolid (LZD) [91]. Here we investigated the potential of *Staphylococcus aureus* strains ATCC 29213 (methicillin-susceptible *S. aureus* MSSA) and ATCC 33591 (methicillin-resistant *S. aureus* MRSA) to develop resistance to TR-700. The spontaneous frequencies of mutation of MSSA 29213 and MRSA 33591 resulting in reduced susceptibility to TR-700 at 2x the MIC were 1.1 × 10^−10^ and 1.9 × 10^−10^, respectively. These values are ~16-fold lower than the corresponding LZD spontaneous mutation frequencies of both strains. Following 30 serial passages in the presence of TR-700, the MIC for MSSA 29213 remained constant (0.5 *μ*g/mL) while increasing eightfold (0.25 to 2.0 *μ*g/mL) for MRSA 33591. Serial passage of MSSA 29213 and MRSA 33591 in LZD resulted in 64- and 32-fold increases in LZD resistance (2 to 128 *μ*g/mL and 1 to 32 *μ*g/mL, resp.). Domain V 23S rRNA gene mutations (*Escherichia coli* numbering) found in TR-700-selected mutants included T2500A and a novel coupled T2571C/G2576T mutation, while LZD-selected mutants included G2447T, T2500A, and G2576T. We also identified mutations correlating with decreased susceptibility to TR-700 and LZD in the *rplC* and *rplD* genes, encoding the 50S ribosomal proteins L3 and L4, respectively.

Resistance to linezolid (LZD) occurs through mutations in 23S rRNA and ribosomal proteins L3 and L4 or through methylation of 23S rRNA by Cfr [92]. Here we report novel L3 mutations, ΔSer145/His146Tyr and ΔMet169-Gly174, cooccurring with *cfr* in LZD-resistant *Staphylococcus aureus* isolates recovered from a hospital outbreak in Madrid, Spain. LZD MIC values (16, 32, or 64 *μ*g/mL) correlated with the presence and severity of the L3 mutation. All isolates had TR-700 (torezolid) MIC values of ≤2 *μ*g/mL.

The crystal structure of the antibiotic drug candidate RWJ-416457 (Figure 30) (systematic name: *N*-{(5*S*)-3-[4-(5,6-dihydro-2*H*,4*H*-2-methylpyrrolo [3,4-*c*]pyrazol-5-yl)-3-fluorophenyl]-2-oxo-1,3-oxazolidin-5-yl}acetamide), C_18_H_20_FN_5_O_3_, which belongs to the first new class of antibiotics discovered in the past 30 years, has been determined at 150 K [93]. Each molecule of this drug donates one hydrogen bond to a neighboring molecule and accepts one hydrogen bond to give (O=C–*R*–N–H*⋯*O=C–*R*–N–H*⋯*)*_n_* linkages along the *b*-axis direction. The compound contains a pyrrolopyrazole ring, which, owing to its uncommon structure, has been shown to have particular effectiveness against multidrug-resistant bacteria.

Locke et al. have reported that staphylococcal resistance to linezolid (LZD) is mediated through ribosomal mutations (23S rRNA or ribosomal proteins L3 and L4) or through methylation of 23S rRNA by the horizontally transferred Cfr methyltransferase [94]. To investigate the structural basis for oxazolidinone activity against LZD-resistant (LZD^r^) strains, we compared structurally diverse, clinically relevant oxazolidinones, including LZD, radezolid (RX-1741), TR-700 (torezolid), and a set of TR-700 analogs (including novel CD-rings and various A-ring C-5 substituents), against a panel of laboratory-derived and clinical LZD^r^ *Staphylococcus aureus* strains possessing a variety of resistance mechanisms. Potency against all strains was correlated with optimization of C- and D-rings, which interact with more highly conserved regions of the peptidyl transferase center binding site. Activity against *cfr* strains was retained with either hydroxymethyl or 1,2,3-triazole C-5 groups but was reduced by 2- to 8-fold in compounds with acetamide substituents.

This phase 4, randomized, double-blind, multicenter trial compared the efficacy and safety of Zyvox with vancomycin in the treatment of nosocomial pneumonia proven to be caused by MRSA, a serious and difficult-to-treat bacterial infection that is resistant to many antibiotics [95]. The study randomized 1,225 patients between 2004 and 2010. The study was designed as a noninferiority study with nested superiority, meaning the primary endpoint would be tested for superiority if it met non-inferiority criteria. In the study, Zyvox was noninferior and statistically superior to vancomycin in achieving both clinical and microbiologic success. The primary endpoint was clinical outcome at end of study in the per-protocol population. Secondary analyses included clinical outcome at end of treatment in the per-protocol population, clinical outcomes in the modified intent-to-treat population at end of study and end of treatment, microbiologic outcomes at end of study and end of treatment in the per-protocol and modified intent-to-treat populations, and safety and tolerability in the intent-to-treat population. Patients were randomized to receive Zyvox IV 600 mg every 12 hours or vancomycin 15 mg/kg every 12 hours over the course of seven to 14 days; vancomycin doses could be titrated at the investigator's discretion based on creatinine clearance and vancomycin trough levels.

## Figures and Tables

**Table 1:** Available marketed formulation of oxazolidinone.

Brand name	Dosage form	Dosage	Company
Infulid	TAB	600 mg	Neiss
Linosept	TAB	600 mg	Micro Eros
Linospan	TAB	600 mg	Cipla
	IV	200 mg/100 mL	
Linox	TAB	600 mg	Unichem
Lizbid	TAB	600 mg	Piramal HC
Linezolid	TAB	600 mg	Glenmark (Integrace)
	IV BAG	600 mg	
Lizomed	TAB	600 mg	Aglowmed
	D-SYR	100 mg	

**Table 2:** 

S. no.	Compound name	Clinical phase	Sponser	Conditions
(1)	Rivaroxaban (BAY59-7939)	phase-III	Bayer's Johnson & Johnson Pharmaceutical Research Development	Deep vein thrombosis (DVT) or Pulmonary embolism (PE)
(2)	Radezolid	phase-II Complete	Rib-X pharmaceutics	Uncomplicated skin infection
(3)	Torezolid	phase-II	Trius Therapeutics	Complicated skin and skin-structure infections (cSSSI)
(4)	DA-7218	phase-III	Dong-A Pharmaceutical	Acute bacterial skin and skin-structure infections (ABSSSI)
(5)	MRX-1	phase-I	MicuRx Pharmaceutical	Bacterial infection
(6)	Radezolid	phase-II	Rib-X pharmaceutics	Community-acquired pneumonia
(7)	PNU-100480	phase- IIa	Pfizer pharmaceutical	Pulmonary Tuberculosis
(8)	RX-1741	phase- II	Rib-X pharmaceutics	Infectious skin disease

**Table 3:** Antimicrobial activity of YC-20 against 522 Gram-positive organisms from clinical samples.

Organism/antimicrobial agent	50% MIC (mg/L)	90% MIC (mg/L)	Range MIC (mg/L)
*Staphylococcus aureus*, MSSA (80)			
YC-20	0.25	0.5	0.06–0.5
Linezolid	0.25	1	0.25–1
Vancomycin	0.5	1	0.25–1
Ampicillin	1.0	2	0.25–4
Cefazolin	0.5	4	0.125–4
evofloxacin	0.125	1	0.06–2

**Table 4:** Antimicrobial activity of Radezolid against 522 Gram-positive organisms from clinical samples.

Bacteria	Linezolid (MICs in mg/L)	Radezolid (MICs in mg/L)
*S. pneumonia*	0.5–2	<0.25
*S. pyogenes*	2–4	0.03–0.125
*E. faecalis*	1–16	<0.25–4
*H. influenza*	2–64	0.25–2
*S. aureus*	2–4	.5–4
*L. pneumophila*	4–16	1–4
*C. trachomatis*	8–16	.5–1

**Table 5:** 

Product name	Furaltadone (Figure 30)
Synonyms	5-(Morpholinomethyl)-3-((5-nitrofurfurylidene)amino)-2-oxazolidinon; 5-morpholinomethyl-3-(5-nitro-2-furfurylidine-amino)-2-oxazolidinone; altabactina; altafur; f-150; furazolin; furazoline; furmethanol
MF	C13H16N4O6
Use	Use in UTI
